# Supplementary material for: Dynamic growth risk of incidentally detected gallbladder polyps–A retrospective, single-center analysis
Source: PLoS One. 2025 Dec 23;20(12):e0337890. doi: 10.1371/journal.pone.0337890 (PMC12725534; doi:10.1371/journal.pone.0337890)
Supplement: Attachment — Submitted filename: Peer Review Report GP study.docx [file pone.0337890.s002.docx]

**Peer Review Report**

**Manuscript Title: Dynamic growth risk of incidentally detected gallbladder polyps – a retrospective, single-center analysis**

**Overall Assessment**

This retrospective study by Heinrich et al. investigates the growth dynamics of incidentally detected gallbladder polyps (GPs) in 253 patients over a median follow-up of 66 months at Hanover Medical School (2001–2020). The findings suggest that most GPs regress or remain stable (median growth rate -0.3 mm/year, 46% undetectable at follow-up), with a low malignancy rate (0.4%, 1 case). The study challenges current guidelines advocating strict surveillance for 6–10 mm GPs, proposing a shift toward individualized management. The topic is clinically significant, and the data provides a foundation for refining surveillance strategies. However, methodological limitations and interpretative gaps necessitate revisions before publication.

**Major Comments**

- **Generalizability and Cohort Composition**
  - Over 63.8% of patients had chronic liver disease (e.g., fatty liver), potentially skewing results and limiting applicability to the general population. Subgroup analyses comparing patients with and without liver disease should be conducted to assess its influence on GP growth behavior.
- **Measurement Variability**
  - Ultrasound-based measurements are prone to operator-dependent variability, exacerbated by the retrospective design. The authors should detail ultrasound protocols, quantify inter- and intra-observer variability, and discuss its impact on growth rate accuracy.
- **Statistical Power for Malignancy Prediction**
  - With only one malignancy case, the study lacks sufficient power to identify risk factors or predict cancer development. This limitation should be prominently highlighted in the abstract and discussion to temper clinical conclusions.
- **Guideline Implications**
  - The critique of current European guidelines for 6–10 mm GPs is compelling but requires a balanced discussion, including cost-effectiveness, patient anxiety, and comparisons with existing evidence to support proposed changes.
- **Data Presentation**
  - Figures (e.g., heatmaps) need clearer labeling and legends. Adding summary visuals like Kaplan-Meier curves for polyp disappearance or bar charts for growth trends would enhance interpretability.

**Minor Comments**

- **Terminology Consistency**
  - Standardize the use of “growth rate (GR)” versus “relative growth rate (RGR)” throughout the manuscript.
- **Ethnicity Reporting**
  - Acknowledge the likely homogenous Caucasian cohort as a limitation, given ethnicity’s potential link to GP malignancy risk.
- **Cholecystectomy Context**
  - Provide details on indications for the two reported cholecystectomies, especially the cancer case (initial size 4.7 mm, growth to 10 mm over 26 months).
- **Reference Updates**
  - Incorporate recent systematic reviews or meta-analyses (if available as of July 2025) to contextualize findings.
- **Language and Clarity**
  - Refine repetitive sentences in the discussion for improved readability.

**Key Implications**

- Supports reconsidering surveillance duration for stable GPs <10 mm.
- Advocates for risk-stratified approaches using baseline imaging (e.g., CT/contrast-enhanced ultrasound).
- Highlights the need for multicenter prospective studies to validate results and explore biomarkers.

**Recommendation :Major Revision**
 The study offers valuable insights into GP natural history, but addressing cohort representativeness, measurement variability, and data presentation will strengthen its clinical applicability and reliability .It can be Impactful but requires addressing limitations to enhance generalizability and validation.
